# The Influence of Spatiotemporal Structure of Noisy Stimuli in Decision Making

**DOI:** 10.1371/journal.pcbi.1003492

**Published:** 2014-04-17

**Authors:** Andrea Insabato, Laura Dempere-Marco, Mario Pannunzi, Gustavo Deco, Ranulfo Romo

**Affiliations:** 1 Department of Information and Communication Technologies, Center for Brain and Cognition, Universitat Pompeu Fabra, Barcelona, Spain; 2 ICREA, Institució Catalana de Recerca i Estudis Avançats, Barcelona, Spain; 3 Instituto de Fisiología Celular-Neurociencias, Universidad Nacional Autónoma de México, México DF, México; 4 El Colegio Nacional, México DF, México; University of Oxford, United Kingdom

## Abstract

Decision making is a process of utmost importance in our daily lives, the study of which has been receiving notable attention for decades. Nevertheless, the neural mechanisms underlying decision making are still not fully understood. Computational modeling has revealed itself as a valuable asset to address some of the fundamental questions. Biophysically plausible models, in particular, are useful in bridging the different levels of description that experimental studies provide, from the neural spiking activity recorded at the cellular level to the performance reported at the behavioral level. In this article, we have reviewed some of the recent progress made in the understanding of the neural mechanisms that underlie decision making. We have performed a critical evaluation of the available results and address, from a computational perspective, aspects of both experimentation and modeling that so far have eluded comprehension. To guide the discussion, we have selected a central theme which revolves around the following question: *how does the spatiotemporal structure of sensory stimuli affect the perceptual decision-making process?* This question is a timely one as several issues that still remain unresolved stem from this central theme. These include: (i) the role of spatiotemporal input fluctuations in perceptual decision making, (ii) how to extend the current results and models derived from two-alternative choice studies to scenarios with multiple competing evidences, and (iii) to establish whether different types of spatiotemporal input fluctuations affect decision-making outcomes in distinctive ways. And although we have restricted our discussion mostly to visual decisions, our main conclusions are arguably generalizable; hence, their possible extension to other sensory modalities is one of the points in our discussion.

## Introduction

Although decision making has been approached from various perspectives, it is generally understood as a complex process involving the comparison of different scenarios and the evaluation of the perceived outcomes in light of one's objectives, as in the paradigmatic example of choosing between different job offers. However, the much simpler tasks, such as those involved in perceptual decision making, have proven useful to investigate the neural basis of decision making. Our main focus is on the recent advances in perceptual decision making as addressed by both neurophysiological data derived from single-cell recordings and modeling studies. To guide this discussion, we have selected a central theme, i.e., the assessment of how the detailed spatiotemporal structure of sensory stimuli affects the perceptual decision-making process. This question allowed us to scrutinize the advances made during the last decade in the neural basis of decision making, while at the same time encompassing a number of research issues that have remained open and are the subject of active debate. These issues include: (i) to understand the role of spatiotemporal input fluctuations in perceptual decision making, (ii) to explore how the current results that were mainly derived from a simple two-alternative choice may extend to scenarios with multiple competing evidences, and (iii) to establish whether different types of spatiotemporal input fluctuations affect decision-making outcomes in distinctive ways.

This review is organized in two parts. First, we give a brief overview of the main neurophysiological results derived from single-cell recordings in monkeys performing a sensory discrimination task. This is followed by a critical revision of the two main theoretical paradigms used to explain these data, namely drift diffusion models (DDM) and attractor neural networks (ANN). Subsequently, recent advances in the field, such as the consideration of multiple alternatives, are reviewed. The section “Spatiotemporal Structure of Noisy Stimuli in Decision Making” constitutes the second part of this work. In this section, we have attempted to pinpoint some aspects that remain unresolved under current experimental and theoretical paradigms and to raise questions that could push our current understanding of the neural basis of decision making further. This will hopefully lead to new experiments being designed and conducted.

### The neurophysiology of decision making

Using different tasks and sensory modalities, various brain areas that encode different stages of the decision-making process have been determined. Beyond deep differences in (among others) stimuli, timing, and motor output, the vast majority of the tasks were based on an 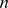-alternative forced-choice (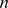
AFC) paradigm. In this paradigm the subjects are always required to commit to a choice among 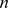 alternatives (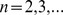), even in the absence of evidence for choosing one of the alternatives at all. Vast amounts of evidence about decision-making processes have been provided in the last decades by studies based on single-unit recordings in monkeys performing 2AFC tasks, either in the somatosensory or the visual domain. Importantly, these studies combine neurophysiological recordings and psychophysical measurements, a type of experimental protocol pioneered in the 1960s by Mountcastle and colleagues [1]–[3].

**Figure 1:**
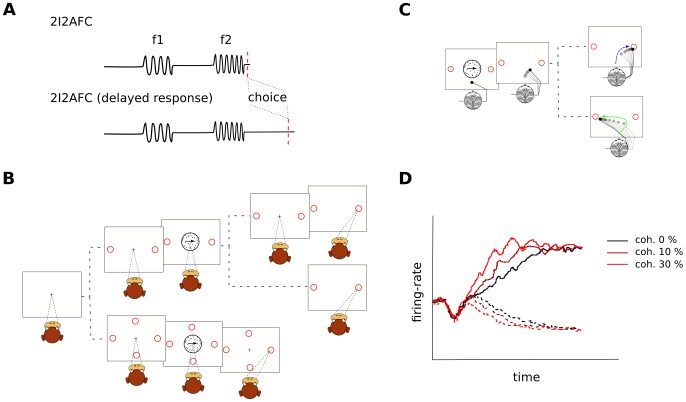
Prevalent experimental paradigms in perceptual decision-making research. (A) Sketch of a vibratory (audio or somatosensory) two-intervals two-alternatives forced-choice (2I-2AFC) discrimination task. In the upper panel, the subject must communicate the decision immediately after reaching it (e.g., [6]), whereas in the bottom panel, the decision should be communicated after a delay period (e.g., [9]). (B) Random dot motion task with two [15], [25] and four [63] targets. The upper branch of the two targets task represents the delayed decision version, where the subject must wait and hold the decision in memory after the stimulus is removed (e.g., [15]). The lower branch represents the RT version, where each trial is terminated by making an eye saccade towards the target whenever the subject reaches a decision. This allows measurement of RT [25]). (C) Random dot motion task with two targets, in which subjects express their decisions through a hand movement. As hand movements are not ballistic, this set-up allows study of the subjects' changes of mind [76]. (D) An example of the kind of neurophysiological data typically obtained in a decision-making experiment. The panel shows a sketch of monkeys' LIP activity during a RDM task for different values of coherence. A complete account of the corresponding real data can be found in [25].

In the vibrotactile frequency-discrimination task in the somatosensory domain, the subject's fingertip is stimulated with a vibrator during two subsequent intervals separated by a delay (see Figure 1A). The subject must decide whether the second stimulation (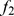) has a higher or a lower frequency than the first one (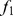) and communicate the decision by pressing one of two buttons [4], [5]. Neurons in the primary somatosensory cortex (S1) have been found to increase their firing rate as a function of the stimulus frequency. During the delay between the two stimulations, the frequency of the first one must be kept in working memory, and neurons in the secondary somatosensory cortex (S2), medial and ventral premotor cortices (MPC, VPC), and dorsolateral prefrontal cortex (dlPFC) were identified to encode stimulus frequency during this period [6]–[8]. When the second stimulation is applied, 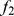 and  must be compared, and some neurons in premotor and prefrontal cortices (and to a minor extent also in S2) encode this comparison in their firing rate, while other neurons encode either 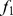 or 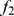. By adding a delay between 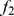 and the response (see Figure 1A, bottom row), Lemus et al. [9] found that the firing of some MPC neurons during this period reflects the comparison between 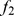 and 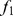, while other neurons still encode either 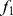 or 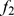, thus suggesting a possible role for this area in the post-decision processing of the choice. Such a role has also been observed in other areas [10]–[13].

As reviewed in [14], the results suggest that the decision-making process is implemented in the brain in a distributed and gradual fashion, and thus, there is no such thing as a single decision locus. This also seems to be the case in the continuous processing relating sensory activity, the formation of a decision variable, and the motor activity, in that the borders separating these stages do not appear to be so clearly demarcated in the primate brain.

In visual discrimination tasks, the great richness of features of our visual experience enables the design of a variety of decision-making tasks, including, but not limited to, the discrimination of motion (e.g., [15], [16]), heading [17], disparity [18], and bar orientation [12], [19]. A prevalent task is random dot motion (RDM) direction discrimination (e.g., [15], [20], see also Figure 1B). In this task subjects look at dots, some of which display random movement while others move coherently in one direction. Subjects must decide which is the direction of coherent motion (even when there is none) and the typical response is made by an oculo-motor movement towards the corresponding visual target. The percentage of dots moving coherently determines the difficulty of the trial. This task allows one to study the various stages of a decision: evidence formation, its integration into a decision signal, holding the decision in memory (in fixed-time experiments), and the commitment to a choice. Neurons in middle temporal area (MT) are tuned to motion and therefore provide the sensory evidence for the decision [21]–[24], whereas lateral intraparietal area (LIP) and frontal eye fields (FEF) were found to integrate the evidence into a decision signal. After stimulus onset, LIP neurons present a dip in firing rate (see Figure 1D). Subsequently the activity varies according to the subject's choice: for stimuli moving towards the response field (RF) of the neuron the firing rate increases, while for movements in the opposite direction the rate decreases. The slope of the ramping correlates with trial difficulty. Both in reaction time (RT) [25] and fixed duration experiments [15], [16], the activity reaches an asymptotic value about 70 ms before saccade initiation, thus suggesting the existence of a decision criterion like the one postulated by diffusion-like models (see next section). Indeed, the results obtained in the visual domain comply well with the view of decision as an integration of sensory evidence until a criterion is reached.

While all the previously discussed results show that several aspects of decision-making processes can be unveiled by analyzing the neuronal recordings in monkeys, our understanding of these phenomena may also be promoted and, indeed, consolidated by directly stimulating the neural system. Following this approach, several studies have demonstrated that electrical microstimulation of areas involved in decision making, both in the somatosensory [26] and in the visual [27]–[29] domain, show similar effects to those observed when the sensory organs receive the stimulation.

### Integration of noisy evidence: Two modeling perspectives

As has been previously stated, 2AFC tasks have been commonly used to investigate decision-making processes. Although several theoretical models have been proposed to explain the available neurophysiological results, most of them [30]–[35] share the fundamental assumption that an integration of noisy evidence over time takes place, thus accumulating such evidence until a decision is made. It is beyond the scope of this review to describe all of these models in detail. Instead we will focus on the two main competing theoretical frameworks, namely the drift diffusion model (DDM) and the attractor neural networks (ANN).

**Figure 2:**
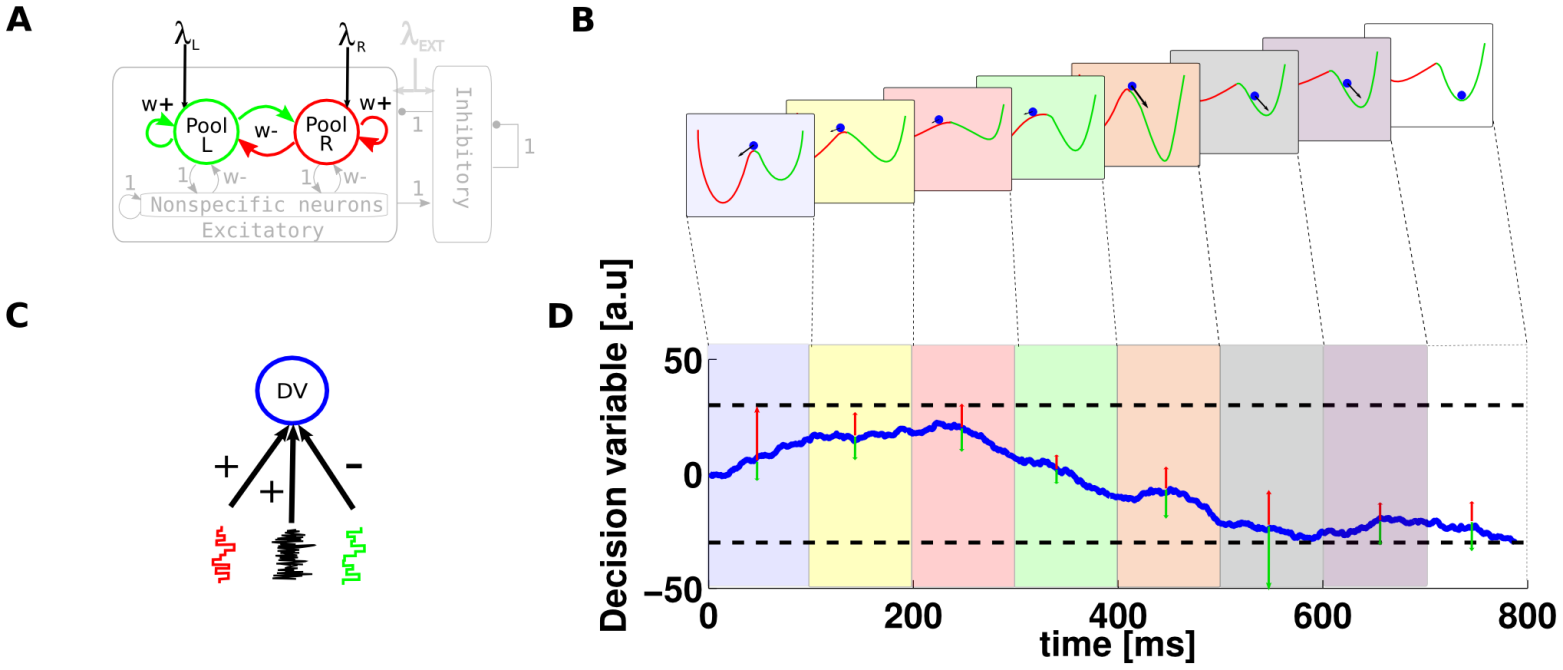
The effect of stimulus fluctuations on behavioral estimates of performance. (A) ANN model sketch. Connectivity parameters can shape the dynamical regime of the network. (B) Representation of the dynamical state of the network. Each panel depicts the hypothetical energy landscape that undergoes modulation due to input fluctuations. The stimulus fluctuations tilt the potential profile, biasing the decision process. (C) Diffusion model sketch. The red and green stair traces represent the time varying drift, whereas the black trace represents the noise. (D) Decision variable dynamics of a DDM stimulated with a variable input (as in panel B), represented by the green (right evidence) and red (left evidence) arrows.

Historically, the DDM [36] was developed first and has been broadly used since then. The equation implementing the DDM in a 2AFC task is based on a continuous variable, 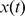, representing the accumulated difference between the two alternatives (see Figure 2C for a sketch). In its simplest implementation, 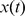 describes a Wiener process and is integrated over time according to:
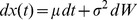
(1)where 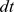 is the accumulated time interval, 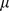 is the evidence to be accumulated (inversely proportional to task difficulty and named drift rate), and 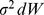 is the so-called noise-diffusion term. The value of 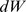 is a number extracted from a normal distribution with zero mean and standard deviation equal to the square root of 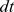. The decision-making process is accomplished when 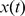 reaches one of two boundaries (see -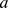/2 or 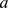/2 in Figure 2D).

The overall RT can be thought of as the sum of the time that 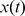 takes to reach the boundary, i.e., the decision time and non-decision time components that account for sensory and motor processing. It is worth noting that several common extensions of the standard DDM have been proposed to specifically account for across-trials variations related to fluctuations in (1) the starting point value, (2) non-decision processing components, and (3) the drift rate. One of the main strengths of the DDM stems from its ease to fit behavioral data [37]. In this respect, DDM has been used to test a broad range of psychophysical hypotheses (e.g., [38]). In particular, DDM accounts well for RT and performance distributions for different task procedures, and for speed-accuracy trade-offs (e.g., with or without time pressure; see [39] for a review). Moreover, it has been shown that DDM can reproduce the shape of the RT distributions both when it is approximately Gaussian [40], [41] (with a model implementing collapsing boundaries) and when it has the usual positive skewness [39], [42] (with fixed boundaries). All of these examples illustrate a situation that is inherent to models that can be flexibly adapted to a large variety of specific situations, i.e., different implementations of a DDM can account for different types of behavior by manipulating certain parameters of the model.

Generally, a number of features that include average and instantaneous drifts, changes in boundaries [40], [41], or the introduction of a leak parameter (hence obtaining a stable Ornstein-Uhlenbeck [OU] velocity process, accounting for an imperfect integration), may be easily added to the simplest DDM versions to accurately reproduce behavioral data. Yet it remains to be seen what fundamental insights are to be extracted from such accurate behavioral accounts. Although, in a way, adding and tuning new parameters may lead to substantial fitting improvements, it is not always the case that it goes hand-in-hand with an enhanced understanding of the fundamental underlying processes.

Furthermore, special care when interpreting the results associated with the exploitation of the DDM fitting capabilities should be taken, for one could be tempted to attribute all causality to one particular parameter (or set of parameters), to the neglect of other causal elements. We should thus bear in mind that naive interpretations of Occam's razor, combined with methods prone to overfitting, can lead to the disregard of certain relevant features [43].

It is worth pointing out that the DDM is a phenomenological model and therefore does not attempt to provide a detailed description of the neural mechanisms that underlie decision making. Nonetheless, a biological motivation for the DDM was recently proposed [35], [44] and we will discuss this later. In contrast to DDM, nonlinear ANN models of spiking neurons crucially seek to provide a biophysically inspired description of these processes. Such ANN models were initially used to explain the neurophysiological basis of other cognitive functions, such as working memory [45]–[47]. Indeed, the observation that, besides decision-related activity, LIP neurons also exhibit persistent activity during delay periods [15] inspired Wang to explore the possibility that ANN of working memory could also explain the integration of stimuli and the formation of perceptual choices [32].

In ANN models the long-term behavior of nonlinear dynamical systems, defined by neural networks of interconnected neurons, is described by so-called *fixed points*. These split the configuration space into basins of attractions. Such basins arise from the initial configuration of the system that leads to the same attractor. In this theoretical framework, 2AFC decision making can be modeled by an attractor network with a minimum of two stable fixed points, which represent the two alternatives. Such a system would display bistability and the transition from an initial configuration towards one of the two stable attractors (i.e., stable unless a sufficiently large perturbation takes the network out of the attractor) would correspond to the decision process.

A type of ANN that is commonly used in decision-making research consists of 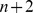 populations (pools) of leaky integrate and fire neurons with common inputs and connectivities, where 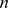 corresponds to the number of choices in 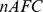 tasks [32], [48]–[50]. The 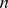 integrators are implemented by pools of excitatory neurons that respond selectively to one of the alternatives and are thought to encode decision-related activity. The other two populations correspond to a homogeneous pool of inhibitory neurons, globally connected to all neurons in the network, and a pool of excitatory neurons, which is not selective of any of the alternatives. The models exhibit recurrent connections between cells from the same selective pool potentiated by a factor 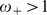 with respect to the baseline connectivity level, and weakened connectivities by a factor 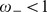 between cells from different selective pools, following the hypothesis of Hebbian plasticity (i.e., synaptic efficacies are modified by neural activity following a training process). This is a key aspect in the formalism of attractor dynamics, which endows the system with the capability to implement a biased competition of the different populations of excitatory neurons that is mediated by inhibition. The competition and cooperation processes thereby established are the basic elements of the underlying neural computations.

Especially during the last decade, which has seen an increase in experimental evidence, both theoretical frameworks have proved successful in accounting for the reported findings. One such example can be found in the case of the previously described motion discrimination task where motion pulses influence both behavior and LIP neural activity, with the later pulses being less relevant than earlier ones [51], [52]. A DDM with a leakage term was able to reproduce this experimental finding, while the time-varying dynamics of the attractor model explained both behavioral and neural data [53], [54].

Although at the expense of reduced biological plausibility, one of the great advantages of DDM over ANN is the fact that DDM is described by a single equation. In contrast, ANNs are endowed with richer dynamics, thus allowing one to model neurophysiological data (i.e., neuronal spiking activity) that may subsequently be used to derive behavior. Nevertheless, the mean-field approach [47] can also reduce the amount of equations of the ANN, thus leading to a formal framework that allows the analytical treatment of dynamical systems. In this approach the number of equations is proportional to the number of different populations of neurons. A further step was made by an approach that combines numerical and analytical methods (i.e., mean-field) to reduce the system to two rate equations; see [55]. Later, Roxin and Ledberg [56] derived a formal relation between the mean-fied reduction of the ANN and a one-dimensional nonlinear diffusion in the proximity of the bifurcation to bistability where the spontaneous state destabilizes. This is a valid reduction for all the winner-takes-all models, which lets one relate the variables of the nonlinear diffusion process to those of the full spiking-neuron model, and thus, to neurobiologically meaningful quantities. Otherwise, Smith [35] takes a different approach to provide a neurophysiological motivation to the DDM. First, he shows that, after initial transient effects, a Wiener process is equivalent to an integrated OU process. As is already well known from the Stein model [57], an integrated OU process can be approximated to a pair of opponent shot noise processes (when their intensity is very high). The link with neurophysiology can be established in that shot noise processes have been used to model neural responses. In a subsequent study Smith and McKenzie [44] provided an alternative analysis that demonstrated how a time inhomogeneous OU velocity process emerges even in the context of a simple recurrent architecture. All in all, the relations between ANN and DDM are complex, mathematically involved, and need to be further analyzed.

### Beyond 2AFC tasks

The study of 2AFC has paved the way for establishing basic principles underlying decision making. But such tasks neglect important aspects inherent to most decisions. However, these aspects can still be considered in highly simplified experimental scenarios such as those used in typical psychophysical or neurophysiological experiments. Such aspects include the consideration of multiple alternatives, the possibility of changing one's mind, and the effect of different sources of neural response variability on decision making. Admittedly, many other aspects, such as those related to subjective experience during decision making (e.g., value-based decision making [58] or confidence-related decision making [59]), have also received notable attention. These aspects are, nonetheless, beyond the scope of this review.

The study of decision making between multiple alternatives was already being addressed from a psychophysical perspective in the 1950s (e.g., [60]). A number of experimental procedures have been used to this end (e.g., absolute identification paradigms [61] and the study of preferential choice [31]). However, only during the last few years have single-cell neurophysiological recordings been becoming available [62]–[65] (see [66] for a review).

It is also worth noting that theoretical attempts to account for multiple choice decision making were already being made in the 1970s and have since been an active area of research [30], [31], [33], [67]–[69]. The first attempts to model multiple choices were made in the context of preferential choice [67], [68], but they failed to reproduce all the experimentally described effects [31]. Later Roe et al. [31] extended the decision field theory of Busemeyer and Townsend [30], which is a kind of sequential sampling model, to account for the multiple preferential choice paradigm. In a subsequent work, Usher and McClelland [33] introduced leakage and nonlinearity in the multiple accumulator model. Building on this work, Bogacz et al. [69] studied the role of nonlinearities and the application of this model to value-based decisions and to Weber's law. The general idea behind it all is that a family of models known as race models [70] (where each target or decision is described by an accumulator that is close in formulation although not mathematically equivalent to DDM [34]) can easily be extended to multiple targets by the simple addition of more integrators.

The first single-cell neurophysiological recordings in a multiple-alternative discrimination task were made by Churchland et al. [63]. The recordings were acquired in monkeys (area LIP) on a two- and four-choice direction-discrimination task, while behavioral results were also being registered. These results have been theoretically modeled in different studies [49], [71], [72]. Beck et al. [71] followed a probabilistic approach with special emphasis on optimality, whereas Furman and Wang [72] and Albantakis and Deco [49] pursued a neurodynamical approach with an emphasis on the detailed biophysical description of the circuitry that underlies decision making.

Of special interest is the situation where multiple choices simultaneously receive evidence, hereafter denoted as multiple competing evidences. Following this experimental paradigm, Niwa and Ditterich [73] tested human participants on a 3AFC version of the RDM task. A key aspect of their experimental setting was the multicomponent RDM stimulus, i.e., a stimulus comprised of up to three coherent motion components instead of just one direction of coherent motion. Thus, the amount of sensory evidence for all three alternatives could be controlled.

It is worth noting that in a subsequent work [74], different DDMs (e.g., with/without leak, lateral/feed-forward inhibition) were tested on these experimental data. This study showed that all models explained the behavioral data equally well. In particular, one of the diffusion models used in that study had a similar architecture to a commonly used, biologically plausible ANN model, that is, with one common inhibitory pool, thus suggesting that a spiking neural network could account for the behavioral data as well.

Later, Bollimunta and Ditterich [64] used the same experimental paradigm with monkeys while recording neurophysiological activity from LIP. Their experimental results suggest that a unique variable in the 3AFC task, the net motion strength (NMS) variable, suffices to predict monkeys' accuracy and RTs. The NMS is defined by the amount of information associated with the highest coherence (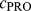) and the average coherence of the second and third components (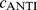) as 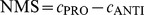. The NMS thus aims to capture all available evidence within a single variable.

Interestingly, these neurophysiological results seem to challenge the class of ANN models that explain well both behavior and neural activity in decision-making tasks [75]. Specifically, Bollimunta and Ditterich suggest that competition cannot be solely mediated by lateral inhibition and they indicate that feedforward inhibition is a necessary component of the neural circuitry that underlies their data. Such conclusions are based on the fact that the firing rates of LIP neurons seem to show an earlier influence of the inhibitory sensory evidence than that driven by the excitatory sensory evidence. In the case of the neurophysiological recordings, the inhibitory sensory evidence (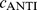) corresponds to the evidence against choosing the target that is in the receptive field (RF) of the neuron being recorded, whereas the excitatory sensory evidence (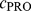) corresponds to that evidence in favor of choosing the target in its RF.

However, in contrast to the conclusions derived from the reported experimental results, it is worth noting that the NMS fails to predict behavioral performance in those cases where the difference between the coherence of the two motion components with less coherently moving dots is large. To illustrate this point, let us consider a situation where the coherence of each motion component is 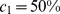, 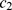 = 45%, and 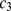 = 5% (NMS = 25). Based on the hypothesis that NMS can be used to predict behavioral performance, this performance should be equivalent to that obtained when 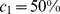, 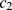 = 25%, and 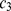 = 25%. Of course, this is clearly not the case. This is relevant because the firing rates of the LIP neurons reported in [64] are pooled on the basis of a variable akin to the NMS previously described. The variable 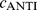 as defined in the context of the neurophysiological recordings may, however, contain large differences between the coherences of the components defining the inhibitory sensory evidence.

It is also worth noting that most studies, both in the DDM and the ANN framework, consider that a decision is made once an established threshold is reached. One may wonder how such a mechanism could accommodate a change of mind. Resulaj et al. [76] addressed this question experimentally by means of a psychophysical RDM task, where human subjects had to indicate the selected choice by moving a handle towards a left or right target. By using continuous hand movements, as opposed to ballistic saccades, changes of mind could (occasionally) be observed in the handle traces (see Figure 1C). Although these findings seem to pose a challenge to ANN (given the previously established stability of the decision-attractors), Albantakis and Deco [50] showed that the attractor picture is entirely consistent with the reported experimental data. This is the case when the system operates close to bifurcation, thus separating a state of categorical decision making from a multistable region. In this region, the existence of an attractor encoding the scenario where all possible alternatives fire at a high rate makes it difficult to reach a decision, thus facilitating changes of mind.

All in all, as has been repeatedly underlined [77], [78], it is of utmost importance to continue the study of decision-making processes beyond the simplest 2AFC experimental paradigm, both from a bottom-up perspective and from a top-down perspective. The bottom-up approach seeks to generate behavioral and neurophysiological predictions derived from biophysically detailed models, whereas the top-down perspective, starting from expected behavior (like DDM optimality or the mathematical assumptions underlying Bayesian inference models), may be adopted to predict low-level implementations. The integration and cross-talk between these two approaches may substantially push forward our current understanding of decision making.

## Spatiotemporal Structure of Noisy Stimuli in Decision Making

The relation between the different research issues that we have addressed in this work becomes more explicit when we consider the central question of how the spatiotemporal structure of sensory stimuli affects the perceptual decision-making process. In order to tackle the various issues that stem from such a question, the RDM discrimination task has received particular attention in this work, due to the large body of relevant experimental results available. Special emphasis has gone to pinpoint some aspects that still remain underconstrained in the current modeling paradigms. Indeed, some of the issues addressed will be of special interest to the modeling community, although definite answers to such questions can only come from the interplay with experimental research.

### Challenges in the investigation of decision processes with multiple competing evidences

When we compare 2AFC to 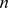
AFC tasks with multiple competing alternatives, it is probably the spatial structure of the RDM stimulus that changes the most. In particular, the fact that each of the multiple alternatives receives its evidence simultaneously adds another level of complexity to the problem. This raises certain interesting issues that we will now explore.

A key aspect when modeling decision-making processes is the definition of the inputs to the decision system. One could, for instance, adopt a simplified phenomenological approach whereby the input to each decision integrator linearly increases with the coherence of the motion component to which the integrator is selective.

When considering a RDM, various parameters can be manipulated [79], [80] and the activity of MT neurons depends on them in different ways. In this task, the activity of MT neurons is commonly regarded (from a modeling perspective) as the most elaborate sensory signal that enters the decisional area LIP. Consequently, it is the signal that has been herein denoted as input to the decisional system.

Of special interest to our discussion are the neurophysiological results, which show that the activity of MT neurons does not linearly depend on the dot density [21], [81], [82] but, rather, shows a divisive normalization effect. However, such activity is arguably linear with regards to coherence [22], [83] in RDM visual stimuli with a single coherent component. Moreover, neurophysiological data available for multiple components [84] suggests that the response to the transparent motion of direction-selective neurons in MT can be “approximated by the scaled sum of their responses to the individual motion components” (see [84], p. 274). On the one hand, this indicates that the linear approximation holds for multiple components. On the other, it implies that one cannot distinguish between two or multiple components if the linear sum of their MT neural activity is the same.

In contrast, based on tests on human participants in a 3AFC version of the RDM task with three coherent motion components, Niwa and Ditterich [73] have provided evidence for a divisive normalization mechanism. In the data of [73] there are three distinct effects that should be accounted for: (1) for 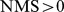 the probability of correct responses decreases when the coherence of the two weaker components increases, (2) for 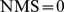 the RTs are faster for increasing coherence of the weaker components, and (3) for 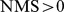 the RTs are slower for increasing coherence of the weaker components. The authors account for these results using a race model with feed-forward inhibition that receives input from a sensory layer implementing a divisive normalization mechanism.

We note here that, although one could be tempted to seek the origin of such effects in the particular architecture or integration method, the behavior of the model is already shaped by the particular divisive normalization of the input layer in [73]. Indeed, for 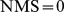 and increasing coherence of the weaker components, the input to the three integrators increases, speeding up the integration process (this is also true when the divisive normalization is substituted by a linear function). When 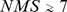 the input to the integrator associated with the strongest component decreases as a function of the coherence of the weaker components. As a consequence, the first passage time increases. Moreover, the input to the integrators associated with the weaker components increases, thus also increasing their probability to reach the threshold first and lowering, in turn, the probability of correct responses.

Therefore, a closer analysis of the signals obtained in the 3AFC task in [73] when applying a divisive normalization reveals the emergence of a structure which already encodes the decision in the input. In addition, a subsequent work by Ditterich [74] has shown that several distinct architectures can explain these data if provided with the same divisive normalization mechanism in the input stage. However as other mechanisms than a divisive normalization of the MT responses could be responsible for the reported behavioral results (as, for example, an elaboration of the signal in higher cortical areas), we would want to argue that further neurophysiological studies are necessary to establish the response of MT neurons to the type of stimuli employed in multiple-choice decision making for simultaneously competing evidences. Furthermore, one should take into account that qualitatively different behavioral results are obtained across species (human [73]; monkeys [64]). From a modeling perspective, the characterization of the input to the decision system might play a critical role in this regard. In brief, we note here that it is not clear how to treat multiple-component stimuli and that further experimental results are needed to shed more light on this issue.

Other questions arise when considering the choice mechanism in relation to multiple alternatives. By choice mechanism, we mean here the way a commitment to a choice is determined in decision-making models. Historically, DDMs used a threshold to terminate the accumulation process [36], and this mechanism is compatible with neurophysiological findings in LIP, as was already explained above. And yet, when facing fixed-time experiments, some investigators disregard the threshold and determine the choice based on the sign of the decision variable alone (e.g., [11], [85]). In ANN models the decision is given by the position of the system in the attractors' landscape. In 2AFC tasks, since the two decision attractors are separated in the 2D space defined by the firing rates of the decision pools, different possible choice mechanisms can be used. The most frequently used is a threshold on the activity of the decision pools (resembling the classical DDM choice mechanism), but a mechanism based on the difference of activity between pools is also sometimes adopted [86], [87]. When considering multiple alternatives, several functions of the state of the integrators could be used (e.g., difference between the two larger accumulators, between the largest and the mean of the others, etc.). Here again more research is necessary to further constrain the models. The experimental paradigm proposed by Niwa and Ditterich [73] whereby different amounts of evidence can be provided to each of the components seems an ideal candidate to shed some light on this issue. In summary, when considering multiple competing evidences it is important to understand how to extend the models initially developed in the context of 2AFC. In this new scenario, questions such as how to set the decision rules and how to combine the evidences provided by the multiple components acquire special relevance.

### The role of spatiotemporal stimulus fluctuations in perceptual decision making

The stochastic behavior that emerges when subjects must make a decision on the basis of uncertain evidence has long puzzled scientists. Much investigation has gone into the mechanisms that underlie the decision-making process when no net evidence for any particular alternative exists (from an experimental point of view see, e.g., [22], [23] and from a theoretical point of view see, e.g., [32]). In this scenario, noise has gained a leading role as a candidate to explain the origin of the stochastic behavior and is, in turn, at the heart of a heated debate in the modeling community.

On the one hand, different theoretical studies [32], [88] have emphasized the role that noise originating in the nervous system can have in decision making. In these approaches, the importance of a noisy representation internal to brain networks in decision-making processes is prominent and such noise is regarded as the ultimate driving factor of the decision-making process [89]. Indeed, it has been suggested that it is precisely such noise that enables probabilistic transitions between different decision states (e.g., [89], [90]). The noise is said to originate from the stochastic spiking times of neurons in Poisson-like spike trains in finite size networks. On the other hand, studies rooted in Bayesian theories have pointed out that in complex tasks the main source of behavioral variability is suboptimal inference, while internal noise would only be playing a minor role [91]. It is worth noting that suboptimal inference works as an amplifier of the noise already present in the system and not as a new source of noise. Suboptimal inference arises when noisy information from several sources must be combined, but an optimal selection of the weights has not been achieved. It is in this regard that the two approaches previously discussed are, in fact, complementary. Note that the final variability of the compound response can never be higher than that corresponding to the source with the highest noise. To achieve a complete understanding of the decision-making process these approaches should be considered jointly. In fact, additional sources of neural variability (e.g., variability due to attentional effects or stimulus fluctuations, among others) should also be investigated. Only in this way, both the contribution of each individual source and their joint effect will be fully characterized.

Of special interest to this discussion is the experimental finding that in a RDM 2AFC task, the trial-to-trial variability in MT neuronal responses is correlated to monkey's behavioral performance [23]. To describe such relations, Britten et al. [23] introduced the choice probability (CP) measure. This is defined as the area under the ROC-curve obtained from the distributions of the firing rates of a neuron (or a population of neurons), given the two possible outcomes: correct or error. Together with the conclusion that a certain level of correlation between the responses of MT neurons with similar preferred directions is required to account for the observed CP [24], this illustrates the importance in perceptual decisions of both variability and correlations in MT neuronal responses [92]. However, this variability might have different origins (e.g., variability emerging at the network level, inherited bottom-up influences such as input fluctuations or suboptimal sensory responses, and top-down modulations).

We will now discuss some of these aspects. More specifically, we will pay special attention to the role of spatiotemporal stimulus fluctuations in perceptual decision making. To this end, we will review previous modeling studies and assess their main implications for our current understanding of the neuronal basis of decision making. A common way to visualize the behavior of a 2AFC ANN model, such as that illustrated in Figure 2A and considered in [32], is by establishing an analogy with the situation in which a potential energy can be defined. The decision-making process is then treated as the evolution of a particle in an energy landscape. In 2AFC this would lead to a double well potential profile (see Figure 2B), where a falling particle in the energy landscape represents the decision variable. We suggest that if the input to the network varies in time, the potential energy in the landscape picture can be thought of as being continuously tilted, thus modulating the depth of the wells. Consequently, the profile may eventually become steeper on one side and momentarily bias the decision, in what is known as a biased-competition scenario. For comparison, Figure 2C shows the schema of a DDM receiving a similar fluctuating input. In the DDM the fluctuations of the drift-rate momentarily push the decision variable towards one of the two boundaries, producing a similar effect to the energy landscape modulation. In order to account for the stimulus fluctuations, one should first find a suitable spatiotemporal characterization of such fluctuations, an aspect that will be addressed below.

#### Encoding the input signals to decision systems

The accurate characterization of neuronal activity from sensory areas in response to the physical stimuli remains a major research issue. This is indeed the case for MT neuronal activity when RDM stimuli are considered. To fully appreciate this aspect, it is worth recalling that trial-to-trial variability in MT neuronal responses were found to correlate with monkey behavioral performance [23]. Thus, a faithful characterization of the MT signal should clearly be sought, in that key aspects of the decision-making processes critically depend on it.

Let us address this issue from the perspective of a phenomenological approach. In particular, let us consider a family of filters that has a long tradition in the motion-perception literature, called the energy motion filters. Some implications derived from their use in decision-making research will be pointed out. The local motion energy associated with visual stimuli moving in opposite directions is calculated by means of two pairs of linear spatiotemporal filters in quadrature. Each pair is responsive for either the coherent motion direction or its opposite direction. Such filters are usually implemented following the seminal work by Adelson and Bergen [93], whereby each directional filter is defined as the sum of two space-time separable filters whose basic components are Gabor spatial filters and multistage low-pass filters with a small amount of inhibition as temporal filters. The temporal impulse response function (derived to account for human motion perception [94]) is rooted in an early work about the dynamics of photo-receptors [95]. Importantly, these functions define spatiotemporal frequency passband filters that are consistent with the responses observed from MT neurons. In particular, Britten et al. [22] found that the motion energy derived from the filters is modulated by coherence in a similar way to that found in MT cells' responses.

Motion energy has been used to characterize the input to decision-making systems in cases when the temporal evolution of the signal acquired special relevance (e.g., [52], [96]). It is worth noting that although this family of filters is endowed with a sound biological motivation, one may wonder whether the characterization offered by them is sufficient to capture all aspects of the MT signals that play a relevant role in decision-making processes. Furthermore, it is clear that the parameters of the filters influence the resulting motion energy [97], but we are still lacking a deep understanding of what the implications are of such changes on predicted behavior. Finally, in order to apply these approaches to the case of multiple targets, a non-trivial extension of the pairs of filters to include further motion directions is required. To the best of our knowledge, this has not been reported in the literature.

#### The effect of spatiotemporal input fluctuations on perceptual decision making

For any particular coherence level in the RDM discrimination task, the visual stimuli contribute a source of variability. This is due to random spatiotemporal fluctuations that vary from trial to trial, thus potentially increasing the variability of MT neuronal responses. Britten et al. [22], [23], in fact, addressed this issue by recording MT neurons while exact replicates of a given stimulus (i.e., the same spatiotemporal structure of the random dot pattern) were repeatedly shown. These studies found no statistical differences in variance-to-mean ratios (VMR) distributions when comparing responses associated with different (non-replicated) and same (replicated) instantiations of the visual stimuli. Hence, they concluded that the fluctuations in these visual stimuli did not contribute to the variance of the recorded MT neuronal responses. Nonetheless, rather than VMR for the complete duration of the stimulus display (i.e., 2 s), one could also consider the Fano factor (FF) of the underlying random process. Indeed, the FF is the ratio between the variance and the mean of a stochastic, whose value is calculated for different time windows. This point was, in fact, addressed by de la Rocha et al. [92] who found, by re-analyzing Britten et al.'s [22] data, that stimulus fluctuations had a substantial effect on the variance of MT neuronal responses for smaller windows (e.g., 125 ms) in the 0% coherence stimuli. In particular, they found significantly larger FF values for stochastic/non-replicated (FF = 1.35, n = 79) versus replicated stimuli (FF = 1.10, n = 45). In contrast, when the complete duration of the stimulus display was considered (i.e., 2 s), 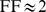 was found in both cases.

From a modeling perspective, Wang [32] had also assessed the effect of small stochastic fluctuations on the decision outcomes. To this end, an ANN model was used. The input to the two decision pools were, in this case, time-dependent, i.e., 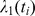 and 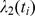. Each 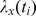 (with 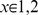) represented the rate of the Poisson process that generated the specific spike trains at the discrete steps of 50 ms. Interestingly, one of the conclusions from this study was that no difference could be observed between the cases when (1) equal rates 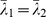 were used as inputs to the network and (2) time-varying rates 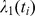 and 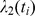 drawn from a Gaussian distribution with mean 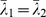 (i.e., same average rate as in the previous case) were used as inputs to the network. Of course, this scenario corresponds to the case when equal evidence is provided to both pools (e.g., 0% coherence).

Indeed, this should be the case when small fluctuations are considered since in both conditions the probability of choosing either direction follows a binomial distribution. Nonetheless, the binomial nature of these processes arises for different reasons. In the first case, both pools have an associated probability 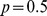 of choosing either right or left. In contrast, in the second case, although the spatiotemporal structure and its associated fluctuations could have biased either option, such bias occurs with a probability 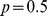 for either direction. Therefore the process overall shares the same stochastic binomial nature and is indistinguishable from the first case. In order to determine whether the particular spatiotemporal structure of the input has in fact a significant impact on behavioral performance, one must compare the probability distribution associated with a given choice under the two following conditions: stochastic stimuli versus replicated stimuli. Clearly, for sufficiently large fluctuations there should be a difference between the two scenarios since one such large perturbation could effectively drive the system towards a particular choice.

All in all, both de la Rocha et al.'s [92] and our discussion regarding Wang's results [32] suggest that further studies are required to accurately assess the role that small spatiotemporal fluctuations play in perceptual decision making. Note, furthermore, that no other processes such as miniature eye movements or attentional shifts are commonly considered when investigating spatiotemporal fluctuations in the stimuli. However, when dealing with behavioral results, attentional processes are likely to play an important role. Therefore, the final impact of such fluctuations in the decision system might be modulated by attentional processes. Ideally, future experimental results where the concerted action of all these variables (e.g., stimulus fluctuations and attentional mechanisms) are controlled for are likely to shed some light onto this issue. Such experiments would contribute to gain fundamental insights on the effects that different sources of shared variability have in decision making.

A very timely work by Brunton et al. [85] constitutes a first effort to define and conduct experiments that aim at characterizing the possible effect of stimuli microstructure variability on decision making. In their experimental paradigm, subjects are presented with trains of pulses, which can be either auditive or visual, and have to judge whether there were more pulses on the right or on the left. By using a stimulus formed of pulses, they can analyze more precisely the role of stimulus fluctuations and distinguish these fluctuations from noise in the diffusion process. To this end, the authors fit a DDM to their behavioral data. In this fitting, the exact timing of each stimulus pulse is considered. Based on the DDM analysis, the authors can discriminate between stimulus fluctuations (pulses) and diffusion noise. The most relevant observation in their own words is, “The dominant source of variability was thus noise in the evidence associated with each incoming pulse” ([85], p. 96), rather than noise in the diffusion process. This is in contrast to Britten et al.'s data [22], [23] but in agreement with the results reported in de la Rocha et al.'s [92], which suggest that stimulus fluctuations could play a significant role in decision making. It is particularly surprising that the authors found a value of the diffusion noise parameter in the DDM indistinguishable from zero in the best fit point. It is, however, possible that their findings are due to the particular paradigm that was used. Note that the stimulus is composed of pulses and strong fluctuations may naturally arise. Whether their results are a general feature of decision-making processes is an interesting issue which deserves further investigation.

To conclude this section, we will briefly recall the possible role that top-down modulations from decisional areas may have on decision making. Interestingly, Nienborg and Cumming [18] have shown that after an initial transient, the CP shows a plateau until the end of the trial. De la Rocha et al. [92] implemented a biologically plausible model accounting for the temporal evolution of the CP shown in [18]. In particular, they introduced a top-down signal from decisional areas into sensory stages. Their results suggest that, to correctly interpret the neurophysiological data, one should take into consideration not only the fluctuations associated with the stimulus but also the fluctuations driven by top-down decisional areas. Top-down modulations of this kind have been demonstrated in multiple experiments [18], [98]–[101] and theoretically interpreted in the ANN framework by [87], [102].

### Do different types of spatiotemporal input fluctuations affect decision making in distinctive ways?

Up until now, we have been claiming that spatiotemporal fluctuations in the stimulus may be an important factor in decision making, when this is considered in appropriate time scales. However, an issue that has not received much attention from either the neurophysiological or the modeling perspective is the impact that different types of stimulus fluctuations have on decision making and how to characterize the neuronal responses to them. To illustrate this issue, we will return to RDM discrimination and pose the following two questions: (1) Do different types of RDM implementations lead to different behavioral results? (2) How, from a modeling perspective, do we take these different types of implementations into account?

In what follows, we will discuss two types of RDM instantiations. Firstly, the case of *white noise* (WN): In the WN instantiation a proportion of points is randomly chosen to move in a direction of motion, i.e., the coherent component. The rest of the dots are randomly relocated within the visual display, thus having a random direction and speed. These dots are the noise component. Secondly, *random direction motion* (RM) (denoted as Brownian motion in [79]): similar to WN, but the points associated with the noise component show the same speed as those that move coherently. A sketch of the two different types of RDM stimuli is shown in Figure 2A, 2B. Interestingly, Pilly and Seitz [79] investigated (from a psychophysical perspective) the ability of human subjects to estimate motion direction in an 8AFC task. In that study, four commonly used RDM implementations (including WN and RM) were considered, and for each implementation some of the parameters defining the RDM visual stimulus were varied. The authors found substantial differences in behavioral performances when certain parameters (e.g., spatial displacement, temporal displacement, or visual contrast) were varied in the various types of stimuli. They related these findings to the spatiotemporal displacement tuning properties of cells in MT. In agreement with the comparative study in a 2AFC task by Scase et al. [80], they reported that subjects discriminated the direction better for RM than WN.

**Figure 3:**
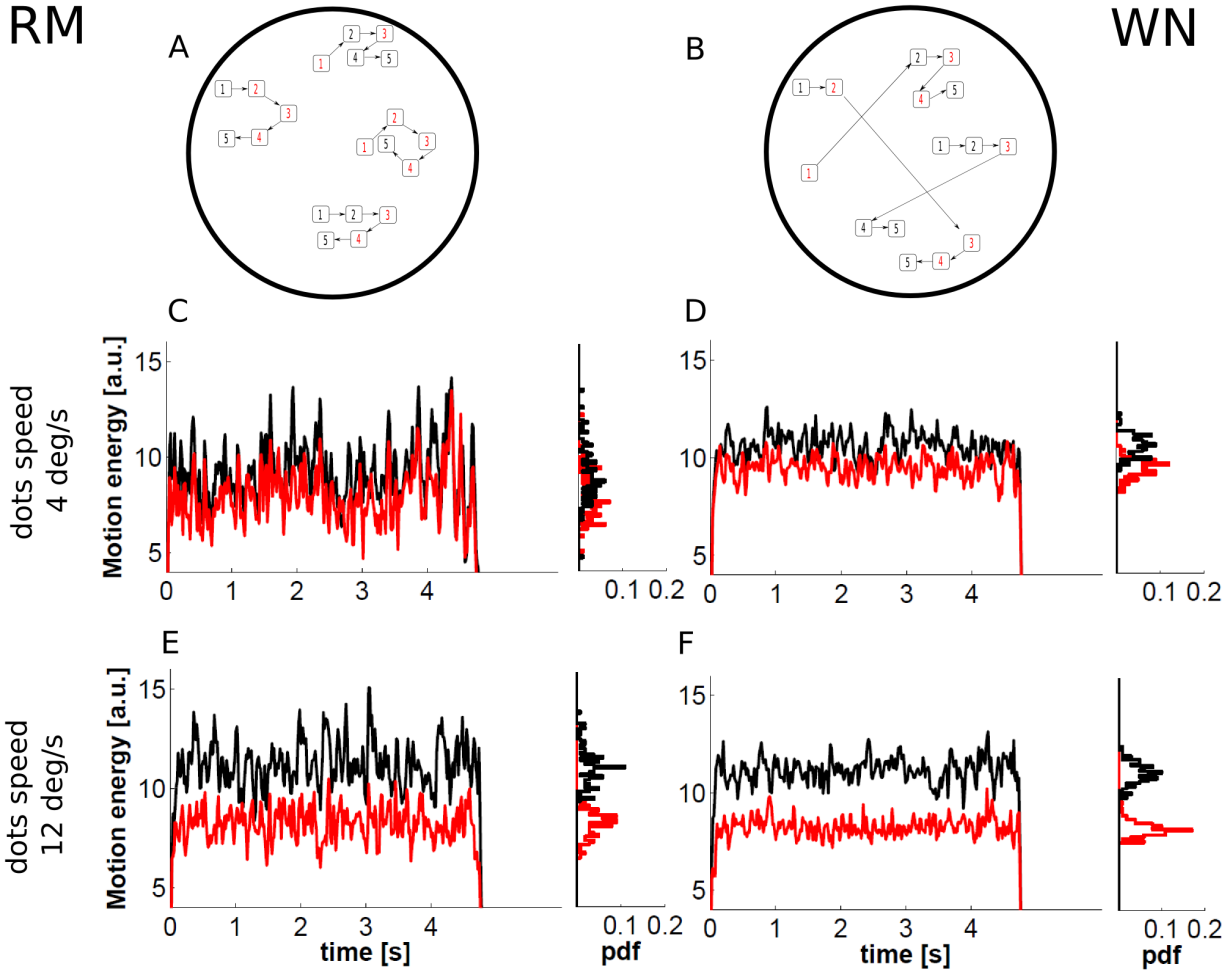
Energy motion associated with different implementations of stochastic stimulus fluctuations. The top panels show illustrations of two implementations of RDM stimulus (see [79] for these and further examples): (A) random motion (RM) and (B) white noise (WN). Panels A and B show sample trajectories of the dots (here represented with a rectangle and a number) for five frames. The dots are moving either towards the right or randomly. The number on each dot represents the frame in which it flashes. At every frame, dots are assigned randomly to either the noise or the signal set. This is represented as red (noise) and black (signal) numbers in the rectangles. See the text for a more detailed description of the algorithms. The four bottom panels show energy motion profiles associated with RM (panels C and E) and WN (panels D and F) for different values of dots speed: middle panels C and D for 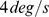 and bottom panels E and F for 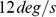. In particular, 2AFC RDM stimuli with motion in two opposite directions and a single coherent component with a coherence level 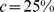 are considered. The black and red curves correspond to the preferred and null direction, respectively. The four histograms show the probability distribution function (pdf) of the average motion energy in a 50 ms time window. In our implementation, the movement of the points was updated every frame (as in [22], [23]), in contrast to every three frames as is common in other studies (e.g., [25], [64], [73]).

Continuing the previous discussion, which illustrates a prominent role of stimulus fluctuations in guiding behavior, one may wonder whether sensory signals in MT (driven by the different types of RDM stimuli) would also reflect differences that are compatible with the psychophysical results reported in [79], [80]. To shed more light on this matter we show in Figure 3 the energy motion profiles of the two types of stimuli in a 2AFC, following the implementation by Zylberberg et al. [96].

Although it is beyond the scope of this review to conduct a systematic study to address all of the available results in [79], and the discrimination task therein used is an 8AFC as opposed to a 2AFC, we would like to pinpoint some inconsistencies that might arise between the experimental results and predictions from current models. In particular, when one compares RDM stimuli with different motion speeds, a large overlap of the energy motion associated with the two directions is found. However, a smaller difference in mean motion energy between the two directions is obtained for the lower speed and RM stimuli. In our illustration, the two velocity of dots considered are 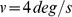 and 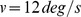. In both cases the stimuli show a motion coherence 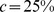 in a single component. This might therefore suggest that, at least for these speeds, RM stimuli leads to inferior performances than WN stimuli, something which is in contradiction with the available results [79], [80]. Moreover, Pilly and Seitz [79] show in one of their experiments that the increase in dot velocity from 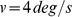 to 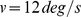 (both in high and low contrast displays) leads to an improvement of motion direction discrimination for WN, but not for RM. However, the energy motion profiles (compare Figure 3C, 3D to Figure 3E, 3F) suggest that both algorithms may benefit from such an increase, as can be derived from an improved separability of the motion energy distributions when the velocity of dots is increased.

As we have previously pointed out, we still lack a deep understanding of how varying the filter parameters (while still conforming to the constraints posed by psychophysical investigations) could influence the resulting motion energy [97]. In this study, motion filters were not intended to function as a complete model of MT activity but only as a first step to characterize the spatiotemporal variability associated with different types of RDM stimuli. The previous discussion suggests that further neurophysiological data considering different types of stimuli would surely shed more light on the suitability of the energy motion filters to characterize MT activity.

## Final Remarks

In this article we have reviewed some of the recent progress made in decision-making research. We deliberately restricted its focus by adopting a computational perspective and have only discussed those studies that investigate the neural basis of decision making. We have critically reviewed both neurophysiological and modeling literature with the purpose of trying to determine the role that the spatiotemporal structure of stimuli has in perceptual decision making. This has allowed us to pinpoint a number of important issues that thus far remain open. Furthermore, we have also raised certain other questions pertinent to the understanding of the mechanisms that underlie the decision-making process. In particular, special attention has gone to the following three issues: (1) the types of responses that are found in sensory areas, when stimuli with multiple competing evidences are taken into consideration (i.e., divisive normalization versus linear summation of independent components), and the new challenges this poses for modeling; (2) the role that fluctuations in the stimulus play as sources of uncertainty within decision making; and (3) the effect on perceptual decisions of different types of noise in the stimulus and how this is treated by certain commonly used phenomenological models.

We have illustrated these aspects by taking examples from visual perception, and more specifically, the RDM discrimination task. The reasons for this were manifold. Firstly, there is a large body of literature that deals with this task. The amount of this literature can only be rivaled by that available on the vibro-tactile frequency discrimination task. However, the vibro-tactile frequency discrimination task generally ignores the spatial dimension inherent to RDM visual stimuli. Moreover, several neurophysiological studies [22], [32] that use RDM stimuli to investigate the role of stimulus fluctuations in decision making exist, and this was an aspect central to our approach. Secondly, for the task used in the literature concerning somatosensory studies in order to make a decision in the vibro-tactile task, the brain must compare two frequencies (i.e., 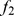 with 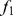). This comparison can only occur after 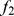 has been applied. Thus, information about 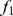 must be held in working memory. This participation of the working memory adds a further complexity to the modeling task. As, however, this element is something that is not shared by other tasks, we have preferred to keep our focus on more elementary aspects.

Nevertheless, we would like to argue that the issues raised in this article may be extended to other sensory modalities. As discussed earlier, Brunton et al. [85] have recently presented research where the role of stimulus fluctuations is assessed in both the auditory and the visual sensory modalities. An extension of their experiments may be easily imagined to include multiple competing evidences (e.g., by presenting trains of pulses coming from multiple directions). Moreover, the vibro-tactile frequency discrimination task could be revisited in a way that complemented the results reported there. Specifically, the periodicity of the signals 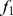 and 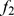 could be slightly broken (i.e., by not keeping the same time interval between two consecutive touches of the fingertip) while maintaining the same overall frequencies. With the advent of these and future experimental results, the influence of the spatiotemporal structure of noisy stimuli in decision making will be further clarified, and with it, our understanding of the mechanisms that underlie the decision-making process.

In this work the neurophysiological data that has been mostly selected and described is that obtained from single-cell recordings in perceptual decision-making experiments. This imposes a particular level of description for our system, which has, in turn, been characterized from different perspectives, i.e., those provided by the various models. Indeed, an effort has been made to critically read the models and their associated interpretations. Our objective has been 2-fold. On the one hand, we have intended to identify those modeling aspects that remain underconstrained and we have pointed out how new experiments might help to better constrain them. On the other hand, we have also aimed to propose specific ways in which the current models could be used to generate new predictions that should be subsequently experimentally tested. It is with this perspective that we want to conclude, i.e., by highlighting the importance of the synergistic interplay between experimental and theoretical work. An excellent example of this joined effort is illustrated by Churchland et al. [103]. The cooperation evidenced by this study can be summarized in three steps. First, experimental data are used to guide and constrain the alternative models. Then those models, which explain equally well the experimental data, are further analyzed to generate predictions that are both testable and crucially different for the various models. This thus allows for the discrimination among the different alternative models. This approach goes beyond the selection criterion of choosing the simplest model. Indeed, as described in [43], the parsimony principle might not be the best way to choose among alternative models when it comes to the biological sciences. What we are recalling here is nothing other than the idea of the virtuous loop, “experiment-model-experiment.”
